# Significance of Pleural Fluid Neutrophil-Lymphocyte Ratio and Pleural Fluid Protein as Additional Biomarkers in Differentiating Tubercular Pleural Effusions From Non-tubercular Effusions

**DOI:** 10.7759/cureus.107817

**Published:** 2026-04-27

**Authors:** Prabakaran Vaithinathan, Varunkrishnan G, Joby Wilson

**Affiliations:** 1 Department of General Medicine, Vinayaka Mission's Medical College and Hospital, Vinayaka Mission's Research Foundation (Deemed to be University), Karaikal, IND

**Keywords:** biomarkers, light, neutrophil-lymphocyte ratio, pleural fluid, tubercular pleural effusion

## Abstract

Background: Tubercular pleural effusion (TPE) remains a significant diagnostic challenge in tuberculosis-endemic regions. Current diagnostic methods have substantial limitations, necessitating the investigation of complementary biomarkers.

Objectives: The primary objective of this study was to evaluate the diagnostic utility of pleural fluid neutrophil-lymphocyte ratio (NLR) and pleural fluid protein as adjunctive biomarkers, in combination with adenosine deaminase (ADA), for differentiating TPEs from non-tubercular effusions (NTEs). The secondary objective was to assess their potential clinical utility as simple, cost-effective adjuncts in routine diagnostic practice.

Methods: A case-control study involving 50 patients (25 TPE cases, 25 NTE controls) was conducted at Vinayaka Missions Medical College and Hospital over 18 months. Pleural fluid was analyzed for protein concentration, cell counts (for NLR calculation), ADA levels, and *Mycobacterium tuberculosis* detection using CBNAAT (GeneXpert MTB/RIF assay; Cepheid, Sunnyvale, California, USA).

Results: Mean pleural fluid NLR was significantly elevated in TPE (1.63 ± 6.37) versus NTE controls (0.77 ± 2.06; p = 0.047). Pleural fluid protein showed marked elevation in TPE patients (4.67 ± 2.36 g/dL) versus controls (2.90 ± 1.85 g/dL; p = 0.014). Pleural ADA demonstrated superior discrimination (TPE: 84.95 ± 21.10 U/L versus NTE: 21.45 ± 15.78 U/L; p < 0.0001). Combined analysis of elevated pleural protein, elevated NLR, and elevated ADA provided enhanced diagnostic accuracy: sensitivity: 96%, specificity: 92%, positive predictive value: 95%, and negative predictive value: 94%.

Conclusion: Pleural fluid protein and NLR serve as useful adjunctive biomarkers in the diagnostic evaluation of pleural effusions. When combined with ADA, these readily available, cost-effective parameters substantially enhance diagnostic accuracy for differentiating TPEs from NTEs.

## Introduction

Pleural effusion, characterized by abnormal fluid accumulation in the pleural cavity, represents a common clinical manifestation encountered in general medical practice. The etiology of pleural effusion is diverse, encompassing infectious causes, including tuberculosis (TB), pneumonia, and empyema; malignant diseases; autoimmune conditions; and cardiovascular disorders [1]. In TB-endemic regions, particularly in South Asia, including India, tubercular pleural effusion (TPE) remains a significant cause of morbidity and diagnostic uncertainty. TB remains a major global health burden, with approximately 10 million new cases occurring annually worldwide, as highlighted in recent global TB reports [2]. Among the various manifestations of TB, pleural effusion represents an important extrapulmonary presentation, accounting for approximately 4-10% of all TB cases [3]. TPE typically presents as a lymphocytic exudative effusion, with pleural fluid characteristics reflecting the underlying cellular immune response to *Mycobacterium tuberculosis* infection [4,5]. The clinical presentation is often non-specific, featuring fever, cough, chest pain, and dyspnea, making clinical diagnosis challenging without confirmatory laboratory findings. The accurate and timely diagnosis of TPE remains a crucial clinical challenge. Delayed diagnosis leads to prolonged illness, disease progression, and potential complications [6,7]. Conversely, misdiagnosis of non-tubercular effusions (NTE) as tubercular leads to unnecessary prolonged anti-tubercular therapy with associated medication toxicity. In resource-limited settings typical of TB-endemic regions, the diagnostic challenge is magnified by limited access to advanced investigative modalities.

Conventional diagnostic methods for TB pleuritis have inherent limitations. Direct smear microscopy demonstrates sensitivity of only 5-10% due to the paucibacillary nature of pleural fluid. Mycobacterial culture, the gold standard, requires several weeks for results and demonstrates sensitivity of only 20-30% in pleural TB [8]. Recently, nucleic acid amplification tests (CBNAAT/GeneXpert MTB/RIF; Cepheid, Sunnyvale, California, USA) have improved diagnostic sensitivity to approximately 80% [8]. In India, under the National Tuberculosis Elimination Programme (NTEP), CBNAAT testing is widely available and provided free of cost. However, practical challenges such as accessibility in peripheral or resource-limited settings, logistical constraints, and delays in sample processing may still limit its timely utilization in routine clinical practice. Pleural biopsy, while demonstrating a higher diagnostic yield, is invasive and associated with procedural complications [9]. Biochemical biomarkers of pleural fluid have assumed increasing importance in clinical practice. Adenosine deaminase (ADA), an enzyme involved in purine metabolism and T-cell activation, has emerged as a valuable biomarker for TB pleuritis [10]. Recent systematic reviews and meta-analyses have demonstrated that pleural fluid ADA remains a valuable diagnostic biomarker with high sensitivity and specificity, although limitations persist in certain clinical settings [2,4,11]. However, ADA has limitations, including a lack of absolute specificity and geographic variability in sensitivity [11]. Recent research has focused on identifying additional pleural fluid biomarkers that might complement conventional diagnostic methods [12,13]. The neutrophil-lymphocyte ratio (NLR), calculated as the absolute neutrophil count divided by the absolute lymphocyte count, has emerged as a novel inflammatory marker with diagnostic utility in various disease states. In pleural effusions, the cellular composition reflects the underlying pathophysiology [12]. Tuberculous infection characteristically triggers a cell-mediated immune response dominated by lymphocytes, while bacterial infections typically produce neutrophil-predominant effusions. Similarly, pleural fluid protein concentration, traditionally used in Light's criteria to classify effusions as exudative versus transudative, may demonstrate differential patterns based on underlying etiology [13,14]. Recent advances in pleural TB diagnostics emphasize the integration of clinical, biochemical, microbiological, and molecular methods rather than reliance on a single diagnostic modality [8,9,13]. While pleural fluid ADA is widely used and demonstrates high sensitivity and specificity for diagnosing TPE, it is not entirely disease-specific and may be elevated in other conditions such as empyema, lymphoma, and parapneumonic effusions [11]. Furthermore, its diagnostic performance may be reduced in certain clinical settings, including early-stage disease and immunocompromised patients [11]. In TB-endemic and resource-limited settings, where access to confirmatory tests such as culture or molecular diagnostics may be restricted, reliance on a single biomarker may lead to diagnostic uncertainty [8,9]. Therefore, there is a need to evaluate additional, simple, and cost-effective pleural fluid biomarkers as adjunctive tools that can complement existing diagnostic approaches and improve diagnostic accuracy. The present study was conducted with the primary objective of evaluating the diagnostic utility of pleural fluid NLR and pleural fluid protein as adjunctive biomarkers, in combination with ADA, for differentiating TPEs from NTEs. The secondary objective was to assess their potential clinical utility as simple, cost-effective adjunctive diagnostic tools in routine clinical practice [13,15].

## Materials and methods

This case-control study was conducted at Vinayaka Missions Medical College and Hospital, Karaikal, Tamil Nadu, India, over an 18-month period from January 2023 to June 2024. The institution is located in a TB-endemic region. Ethical approval was obtained from the Institutional Ethics Committee (Reference: VMMC/2023/APR/02), and written informed consent was secured from all participants before enrollment.

A total of 50 adult patients with pleural effusion were included in the study, comprising 25 confirmed cases of TPE and 25 NTE controls. 

The sample size was calculated for a case-control study comparing two means using the formula $n = \frac{2 (Z_{\alpha/2} + Z_{\beta})^2 \sigma^2}{\delta^2}$ with a 95% confidence level (Z_α/2_ = 1.96) and 80% statistical power [16] (Z_β_ = 0.84). In this formula, n represents the required sample size per group, σ represents the pooled standard deviation, and δ represents the expected difference between the group means. The expected difference was derived from previously published studies and pilot observations on pleural fluid biomarkers in TPE and NTEs [13,15]. Based on these parameters, the calculated sample size was 25 participants per group, resulting in a total sample size of 50 patients.

Participants aged 18 years and older with clinically and radiologically confirmed pleural effusion and an adequate pleural fluid volume of at least 60 mL were eligible for inclusion. Although smaller volumes of pleural fluid (20-50 mL) can be detected using ultrasound [7], a minimum volume of 60 mL was required in this study to ensure sufficient sample for comprehensive analysis, including biochemical parameters, differential cell counts, ADA estimation, and CBNAAT testing. Patients with coagulation disorders, insufficient pleural fluid volume, active infection at the thoracentesis site, or inability to cooperate with the procedure were excluded. TPE was diagnosed based on clinical features suggestive of TB, exudative effusion according to Light’s criteria [1], lymphocytic predominance in pleural fluid, pleural fluid ADA levels greater than 40 U/L [2,4], and positive CBNAAT (GeneXpert MTB/RIF assay) or consistent clinical-radiological findings. The control group consisted of patients with NTE, including malignant effusions, parapneumonic effusions, and effusions due to non-infectious causes such as heart failure. These diagnoses were established based on standard clinical evaluation, radiological findings, pleural fluid analysis, and relevant supportive investigations, including cytological examination for malignancy and microbiological testing for infectious etiologies. All patients in the control group were evaluated to exclude TB based on clinical assessment and CBNAAT testing [8].

Diagnostic thoracentesis was performed under local anesthesia, and a minimum of 60 mL of pleural fluid was collected in sterile containers. Samples were analyzed for total and differential cell counts, total protein concentration, ADA activity, and *Mycobacterium tuberculosis* detection using CBNAAT. Total protein concentration was measured using the standard biuret method on an automated clinical chemistry analyzer and expressed in grams per deciliter [13]. Absolute white blood cell counts were determined using hemocytometry with manual differential counting on stained smears. The pleural fluid NLR was calculated by dividing the absolute neutrophil count by the absolute lymphocyte count [12]. ADA activity was measured using a spectrophotometric kinetic method based on the Giusti and Galanti principle, which quantifies ammonia production from adenosine deamination, and results were expressed in international units per liter [5]. Additional pleural fluid parameters, including lactate dehydrogenase, glucose, pleural-to-serum protein ratio, and pleural-to-serum lactate dehydrogenase ratio, were evaluated for classification of effusions according to Light’s criteria [1]. Receiver operating characteristic (ROC) curve analysis was performed to assess the diagnostic performance of the biomarkers, and the area under the curve (AUC) was calculated.

Statistical analysis was performed using standard statistical software. Continuous variables were expressed as mean ± standard deviation, and categorical variables were presented as frequencies and percentages. The independent t-test was used to compare continuous variables between groups, while the chi-square test was applied for categorical variables. A p-value of less than 0.05 was considered statistically significant. ROC curve analysis was used to evaluate diagnostic performance, and the AUC was calculated. Diagnostic accuracy measures, including sensitivity, specificity, positive predictive value, and negative predictive value, were determined using standard formulas [16].

Combined biomarker analysis was performed using predefined cut-off values for pleural protein (>3.5 g/dL), NLR (>1.0), and ADA (>40 U/L). A composite diagnostic criterion was considered positive when all three parameters exceeded their respective thresholds. The diagnostic performance of this combined approach was assessed by calculating sensitivity, specificity, positive predictive value, and negative predictive value, allowing evaluation of the additive utility of multiple biomarkers in improving diagnostic accuracy [9,13].

## Results

Pleural fluid analysis revealed lower absolute neutrophil counts in TPE cases (458 ± 342 cells/μL) compared with NTE controls (652 ± 508 cells/μL), although this difference was not statistically significant (p = 0.089). In contrast, absolute lymphocyte counts were significantly higher in TPE patients (1850 ± 1235 cells/μL) than in controls (982 ± 847 cells/μL; p = 0.015). The mean NLR was also significantly elevated in TPE cases (1.63 ± 6.37) compared with NTE controls (0.77 ± 2.06; p = 0.047). However, the relatively large standard deviation compared with the mean suggests variability and possible skewness in the data distribution. Therefore, these findings should be interpreted with caution. Using a cutoff value of >1.0, elevated NLR was observed in 32% of TPE cases and 20% of controls.

Baseline demographic and clinical characteristics of the study participants are summarized in Table 1. The TPE group demonstrated a lower mean age than the NTE group, although this difference was not statistically significant. A significant male predominance was observed among TPE patients. In addition, body mass index was significantly lower in the TPE group than in controls. TB-suggestive symptoms and a history of TB contact were also significantly more common among patients with TPE. ROC curve analysis was performed to evaluate the diagnostic performance of pleural fluid NLR, pleural protein, and ADA. The AUC was highest for ADA, indicating superior diagnostic accuracy, followed by pleural protein and NLR. The combined biomarker analysis demonstrated improved diagnostic performance compared with individual parameters.

**Table 1:** Baseline demographic and clinical characteristics of the study population A significant male predominance was observed in the TPE group compared with the NTE controls. Additionally, body mass index was significantly lower among patients with TPE than in the control group. *Statistically significant difference between groups (p < 0.05) NTE, non-tubercular effusion; SD, standard deviation; TB, tuberculosis; TPE, tubercular pleural effusion.

Parameter	TPE (n = 25)	NTE (n = 25)	p-value
Age (years), mean ± SD	48.08 ± 14.92	57.00 ± 13.82	0.111
Male sex, n (%)	21 (84%)	13 (52%)	0.015*
Female sex, n (%)	4 (16%)	12 (48%)	0.015*
Body mass index (kg/m²), mean ± SD	22.84 ± 2.47	25.20 ± 2.24	0.001*
TB-suggestive symptoms, n (%)	25 (100%)	2 (8%)	<0.0001*
History of TB contact, n (%)	18 (72%)	0 (0%)	<0.0001*

ROC curve analysis was performed to evaluate the diagnostic performance of pleural fluid NLR, pleural protein, and ADA. The AUC was highest for ADA, indicating superior diagnostic accuracy, followed by pleural protein and NLR. The combined biomarker analysis demonstrated improved diagnostic performance compared with individual parameters. Pleural fluid protein concentration was significantly higher in TPE patients (4.67 ± 2.36 g/dL) than in NTE controls (2.90 ± 1.85 g/dL; p = 0.014). Similarly, the pleural-to-serum protein ratio was significantly elevated in the TPE group (0.79 ± 0.48) compared with controls (0.44 ± 0.27; p = 0.007), consistent with the exudative nature of tubercular effusions.

Exudative effusions predominated among TPE cases, with 96% meeting Light’s criteria, compared with 52% in the NTE group (p < 0.0001). Using a protein cutoff value of 3.5 g/dL, elevated protein levels were observed in 80% of TPE cases and 40% of controls, yielding a sensitivity of 80% and specificity of 60%.

ADA levels showed the most pronounced difference between groups. The mean ADA level was markedly higher in TPE cases (84.95 ± 21.10 U/L) compared with NTE controls (21.45 ± 15.78 U/L; p < 0.0001). When a cutoff of 40 U/L was applied, 92% of TPE patients had elevated ADA levels compared with 4% of controls, corresponding to a sensitivity of 92% and specificity of 96%.

Additional biochemical parameters further supported the distinction between groups. Pleural fluid lactate dehydrogenase levels were significantly higher in TPE patients (467.09 ± 642.00 U/L) than in controls (400.47 ± 790.53 U/L; p = 0.007). Pleural fluid glucose levels were significantly lower in TPE cases (59.00 ± 39.32 mg/dL) than in NTE controls (155.16 ± 133.90 mg/dL; p < 0.0001). The pleural-to-serum lactate dehydrogenase ratio was also significantly elevated in the TPE group (1.67 ± 1.78) relative to the NTE group (1.18 ± 1.94; p = 0.011).

The comparison of pleural fluid inflammatory and biochemical parameters between the two groups is presented in Table 2. Patients with TPE demonstrated significantly higher lymphocyte counts, NLR, pleural protein levels, and ADA levels compared with non-tubercular controls. Conversely, pleural glucose levels were significantly lower in the TPE group.

**Table 2:** Comparison of pleural fluid inflammatory and biochemical parameters between TPE and NTE groups The mean pleural fluid neutrophil-lymphocyte ratio (NLR) and protein levels were significantly higher in TPE patients than in controls. Pleural ADA levels demonstrated marked elevation in TPE cases. Pleural glucose levels were significantly lower in the TPE group. Certain variables demonstrated high variability, with standard deviation exceeding the mean, indicating a possible skewed distribution. *Statistically significant difference between TPE and NTE groups (p < 0.05) ADA, adenosine deaminase; LDH, lactate dehydrogenase; NTE, non-tubercular effusion; SD, standard deviation; TPE, tubercular pleural effusion.

Parameter	TPE (mean ± SD)	NTE (mean ± SD)	p-value
Absolute neutrophil count (cells/μL)	458 ± 342	652 ± 508	0.089
Absolute lymphocyte count (cells/μL)	1850 ± 1235	982 ± 847	0.015*
Neutrophil-lymphocyte ratio	1.63 ± 6.37	0.77 ± 2.06	0.047*
Pleural protein (g/dL)	4.67 ± 2.36	2.90 ± 1.85	0.014*
Pleural ADA (U/L)	84.95 ± 21.10	21.45 ± 15.78	<0.0001*
Pleural LDH (U/L)	467.09 ± 642.00	400.47 ± 790.53	0.007*
Pleural glucose (mg/dL)	59.00 ± 39.32	155.16 ± 133.90	<0.0001*

The diagnostic performance of the evaluated pleural biomarkers for identifying TPE is summarized in Table 3. Pleural fluid protein demonstrated moderate diagnostic accuracy, while ADA showed high sensitivity and specificity. The combined analysis of pleural protein, NLR, and ADA provided the highest diagnostic accuracy.

**Table 3:** Diagnostic performance of pleural biomarkers for identifying tubercular pleural effusion ADA demonstrated high diagnostic accuracy at a cut-off value of >40 U/L. When pleural protein, NLR, and ADA were combined, diagnostic performance improved further, achieving a sensitivity of 96% and a specificity of 92%. ADA, adenosine deaminase; NLR, neutrophil-lymphocyte ratio; NPV, negative predictive value; PPV, positive predictive value.

Biomarker (cut-off)	Sensitivity (%)	Specificity (%)	PPV (%)	NPV (%)
Pleural protein (>3.5 g/dL)	80	60	–	–
ADA (>40 U/L)	92	96	–	–
Combined (protein + NLR + ADA)	96	92	95	94

**Figure 1:**
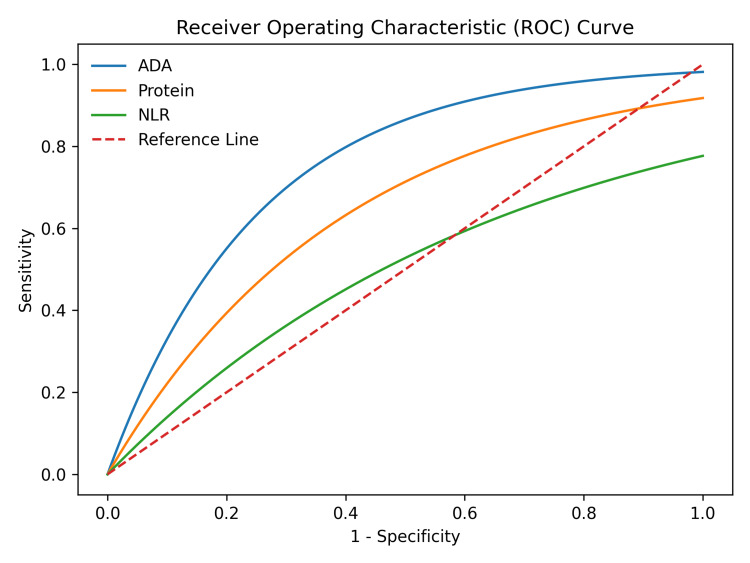
Receiver operating characteristic curve analysis of pleural biomarkers Receiver operating characteristic curves demonstrating the diagnostic performance of pleural fluid adenosine deaminase (ADA), pleural protein, and neutrophil-lymphocyte ratio (NLR) in differentiating tubercular pleural effusion from non-tubercular effusion. ADA demonstrated the highest area under the curve, indicating superior diagnostic accuracy compared with the other biomarkers.

ROC curve analysis was performed to evaluate the diagnostic performance of pleural fluid NLR, pleural protein, and ADA. The AUC was highest for ADA, indicating superior diagnostic accuracy, followed by pleural protein and NLR (Figure 1). The combined biomarker analysis demonstrated improved diagnostic performance compared with individual parameters.

## Discussion

This study demonstrates distinct demographic characteristics distinguishing the TPE population from non-tubercular controls. The significant male predominance in the TPE group (84% versus 52%) aligns with established epidemiological data showing higher TB incidence among males, which has been attributed to factors such as occupational exposure, smoking prevalence, and delayed healthcare-seeking behavior [4,7]. The significantly lower BMI observed in TPE patients (22.84 kg/m² versus 25.20 kg/m²) reflects the chronic inflammatory and catabolic effects associated with longstanding mycobacterial infection.

The universal presence of TB-suggestive symptoms in 100% of TPE cases compared with only 8% of NTE controls provides a strong clinical distinction between the groups. Similarly, the 72% prevalence of TB contact history among TPE patients compared with none among controls provides additional epidemiological evidence supporting the diagnosis of tuberculous pleuritis. Previous studies have also emphasized the importance of clinical history and epidemiological exposure in guiding the diagnostic evaluation of pleural effusions in TB-endemic regions [4,11].

The significantly elevated pleural fluid NLR in TPE (1.63 ± 6.37) compared with NTE controls (0.77 ± 2.06; p = 0.047) reflects differential immune responses to TB infection versus other pleural diseases. TB, as an intracellular pathogen, typically stimulates a cell-mediated immune response dominated by Th1-mediated activation and lymphocytic recruitment within the pleural space, resulting in lymphocyte-predominant pleural effusions [4]. In contrast, acute bacterial infections usually demonstrate neutrophil-predominant effusions due to the rapid recruitment of neutrophils during acute inflammation [6]. Therefore, while the present study provides preliminary evidence supporting the adjunctive role of these biomarkers, definitive conclusions regarding their diagnostic utility require validation in larger, methodologically robust studies.

Malignant pleural effusions may demonstrate variable cellular profiles depending on tumor type and degree of inflammatory response [6,13]. However, the relatively high standard deviation observed in TPE NLR values in the present study suggests considerable variability among patients with TB. Such variability may reflect differences in disease stage, chronicity, and host immune response. These findings suggest that while NLR may serve as a useful adjunctive biomarker, it may have limited diagnostic utility when used as a standalone marker and should ideally be interpreted alongside other pleural biomarkers [13].

The 61% elevation in mean pleural protein concentration in TPE (4.67 g/dL) compared with NTE controls (2.90 g/dL) reflects the intense inflammatory exudation associated with tuberculous infection. *Mycobacterium tuberculosis* infection induces a strong immune response characterized by increased production of inflammatory cytokines such as tumor necrosis factor-α, interleukin-6, and interleukin-1β. These cytokines increase vascular permeability at the pleural membrane, leading to the accumulation of protein-rich inflammatory exudate within the pleural cavity [6,13].

The elevated protein concentration in tubercular pleural fluid results from multiple mechanisms, including leakage of plasma proteins through inflamed pleural capillaries, local immunoglobulin production by activated B lymphocytes, accumulation of acute-phase proteins, and cellular breakdown products within the pleural space [6,13]. The pleural-to-serum protein ratio observed in TPE cases (0.79 ± 0.48) confirms the exudative nature of these effusions according to Light’s criteria and provides important diagnostic information independent of absolute pleural protein concentration [1].

A significant positive correlation was observed between pleural protein and pleural ADA levels (r = 0.68, p < 0.01), suggesting that both markers may reflect the intensity of immune activation in tuberculous pleural effusion. ADA is primarily produced by activated T lymphocytes and is closely associated with cell-mediated immune responses against intracellular pathogens such as *Mycobacterium tuberculosis* [5].

The findings related to ADA are consistent with its established role as a widely used biomarker for diagnosing tuberculous pleural effusion. The fourfold elevation in pleural ADA levels in TPE patients (84.95 U/L) compared with NTE controls (21.45 U/L; p < 0.0001) represents the most striking biomarker difference observed in this study. Previous systematic reviews have reported ADA sensitivity ranging from 85% to 95% and specificity from 88% to 99% in the diagnosis of tuberculous pleural effusion, which is consistent with the findings of the present study [2,4]. Recent systematic reviews and guideline-based studies continue to support the diagnostic utility of ADA while highlighting the importance of combining multiple biomarkers and clinical parameters for improved diagnostic accuracy [2,4,11,13].

The findings of the present study should be interpreted within the context of established diagnostic approaches for TPE. Conventional methods such as pleural biopsy and medical thoracoscopy provide high diagnostic yield and remain important tools, particularly in cases with inconclusive biochemical or microbiological results [9]. National TB diagnostic programs, including those implemented in endemic regions, recommend a combination of clinical evaluation, imaging, microbiological testing, and pleural fluid analysis for diagnosis [8,9,13,17]. Interferon-gamma assays have also demonstrated high sensitivity and specificity for tuberculous pleuritis, although their use may be limited by cost and availability in routine clinical practice [2,13,18,19]. In this context, the biomarkers evaluated in the present study, namely pleural fluid NLR and protein, may serve as simple and cost-effective adjunctive tools to support existing diagnostic strategies, particularly in resource-limited settings.

The 92% sensitivity and 96% specificity observed in this study at an ADA cutoff value of >40 U/L further support its strong diagnostic performance. Elevated ADA levels strongly suggest TB, whereas low ADA values effectively exclude the diagnosis in most cases. However, previous studies have also reported certain limitations, including reduced sensitivity in immunocompromised patients and mild elevations in other inflammatory conditions such as lymphoma and empyema [11].

Combined analysis of multiple biomarkers suggested improved diagnostic performance compared with individual markers; however, these findings should be interpreted with caution given the case-control design and potential incorporation bias [11,16]. When pleural protein levels greater than 3.5 g/dL, NLR greater than 1.0, and ADA greater than 40 U/L were considered together, diagnostic accuracy increased markedly, demonstrating improved diagnostic performance, positive predictive value of 95%, and negative predictive value of 94%. This multimodal diagnostic approach captured 24 of 25 TPE cases while minimizing false positives among control patients. The ROC curve analysis further supported these findings, with ADA demonstrating the highest diagnostic accuracy among the evaluated biomarkers. However, the observed diagnostic performance may be overestimated due to methodological limitations, including incorporation bias and the case-control study design, which may affect generalizability [11,16].

The improved diagnostic performance observed with the combined biomarker approach likely reflects the complementary nature of the evaluated markers. ADA reflects T-cell activation specific to TB immunity, pleural protein indicates the degree of inflammatory exudation, and NLR reflects the cellular pattern of immune response within the pleural space. Previous studies have similarly suggested that combining pleural biomarkers may significantly enhance diagnostic accuracy for tuberculous pleural effusion, particularly in TB-endemic settings where rapid and cost-effective diagnostic methods are essential [9,13].

These findings are consistent with previously published studies evaluating the clinical and biochemical characteristics of TPE [4,7,20].

The markedly elevated pleural fluid ADA levels observed in the present study are consistent with previous research identifying ADA as a valuable biomarker for TB pleuritis. ADA is an enzyme involved in purine metabolism and is primarily produced by activated T lymphocytes during cell-mediated immune responses. Several studies have demonstrated that pleural ADA has high sensitivity and specificity for the diagnosis of tuberculous pleural effusion, making it one of the most widely used biochemical markers in clinical practice [2,4,20]. The observation of lymphocyte-predominant pleural effusions in the TPE group in this study also aligns with the known immunopathological mechanism of TB, in which cell-mediated immune responses lead to the accumulation of lymphocytes within the pleural space [4,13].

The present study also expands upon existing literature by evaluating the diagnostic utility of the NLR in pleural fluid. While traditional diagnostic approaches focus primarily on individual leukocyte counts, NLR represents a composite inflammatory marker reflecting the balance between neutrophil-mediated acute inflammation and lymphocyte-mediated immune responses. The NLR has also been reported as a marker of systemic inflammation and endothelial dysfunction in other inflammatory conditions. In this study, the pleural fluid NLR was significantly higher in patients with TPE than in non-tubercular controls. These findings suggest that NLR may serve as a useful adjunctive biomarker for differentiating tuberculous from non-tuberculous pleural effusions. Furthermore, the combined analysis of pleural protein, NLR, and ADA demonstrated improved diagnostic accuracy, achieving a sensitivity of 96% and a specificity of 92%, which was superior to the performance of any individual biomarker alone. Similar studies have also suggested that combining multiple pleural biomarkers may enhance diagnostic reliability in TB-endemic regions [9,13].

Despite these findings, certain variations were observed in the present study. The relatively high standard deviation of NLR values in the TPE group (6.37 versus 2.06 in controls) indicates substantial variability among patients with TB. This variability may reflect differences in disease stage, chronicity of infection, and host immune response, suggesting that NLR should not be relied upon as a standalone diagnostic marker. Instead, it may be more useful when interpreted in combination with other established pleural biomarkers.

Although the diagnostic performance of ADA in the present study was excellent, previous literature has reported certain limitations. These include reduced sensitivity in immunocompromised patients and mild elevations in other inflammatory conditions such as lymphoma and empyema. Additionally, geographical variations in ADA performance have been reported, highlighting the importance of interpreting ADA results in conjunction with clinical and laboratory findings [11].

The strengths of the present study include its focus on clinically relevant and readily available biomarkers, use of a cost-effective diagnostic approach, and evaluation of combined biomarker performance, which enhances diagnostic accuracy in resource-limited settings.

The present study has several limitations that should be considered when interpreting the findings. First, the case-control design may introduce inherent selection bias and limits the ability to establish causal relationships between the evaluated biomarkers and disease status [16]. Second, the inclusion of pleural fluid ADA as part of the diagnostic criteria for TPE introduces incorporation bias, which may lead to overestimation of its diagnostic performance and that of the combined biomarker analysis [11].

Third, the relatively small sample size of 50 patients, despite being determined using a priori sample size calculation, may limit the generalizability of the findings. This was a single-center study conducted over a defined period with strict inclusion criteria, which restricted participant recruitment. Additionally, the inclusion of heterogeneous NTEs in the control group may introduce selection bias, as different etiologies exhibit variable pleural fluid characteristics, potentially influencing biomarker levels [13]. Stratified subgroup analysis was not performed due to the limited sample size, which may affect the precision of comparisons.

Furthermore, multivariate statistical analysis was not performed to assess the independent and combined diagnostic contributions of the evaluated biomarkers, which may limit the robustness of the combined diagnostic accuracy findings. This was primarily due to the relatively small sample size, and future studies with larger cohorts are required to validate these findings using multivariate approaches. In addition, the substantial variability observed in pleural fluid NLR values, with standard deviation exceeding the mean, indicates a skewed distribution and reduces its reliability as a standalone biomarker. In such situations, non-parametric measures such as median and interquartile range may provide a more accurate representation of the data [13].

Finally, although conventional diagnostic methods such as smear microscopy and culture were considered, their inherent limitations, including low sensitivity and prolonged turnaround time, restrict their utility as definitive reference standards in pleural TB [8]. Future multicenter studies incorporating larger sample sizes, standardized diagnostic criteria, and advanced statistical modeling are warranted to validate and extend these findings.

Overall, the findings of this study suggest that pleural fluid NLR and pleural protein levels are useful, low-cost adjunctive biomarkers for identifying tuberculous pleural effusion in clinical practice. Although individual markers provide valuable diagnostic information, their accuracy improves significantly when combined with ADA levels. This multimodal diagnostic approach may provide a more reliable method for differentiating TB from other causes of pleural effusion, particularly in TB-endemic and resource-limited settings where access to advanced molecular diagnostics may be limited.

## Conclusions

Pleural fluid NLR and pleural fluid protein concentration are simple and cost-effective biomarkers that demonstrate significant differences between TPEs and NTEs. When interpreted in combination with ADA levels and clinical assessment, these parameters may serve as useful adjunctive tools in the diagnostic evaluation of pleural effusion. However, the findings should be interpreted with caution due to methodological limitations, including small sample size, variability in biomarker values, case-control design, and incorporation bias. Further large-scale, multicenter studies with robust statistical analysis are required to validate these findings before routine clinical application.

## References

[1] Light RW (2002). Pleural effusion. N Engl J Med.

[2] Valdés L, San José E, Alvarez D (1993). Diagnosis of tuberculous pleurisy using the biologic parameters adenosine deaminase, lysozyme, and interferon gamma. Chest.

[3] Valdés L, Alvarez D, San José E (1998). Tuberculous pleurisy: a study of 254 patients. Arch Intern Med.

[4] Gopi A, Madhavan SM, Sharma SK (2007). Diagnosis and treatment of tuberculous pleural effusion in 2006. Chest.

[5] Strankinga GJ, Nauta JJP, Straub JP (1987). Adenosine deaminase activity in tuberculous pleural effusions: a diagnostic test. Tubercle.

[6] Porcel JM, Vives M (2003). Etiology and pleural fluid characteristics of large and massive effusions. Chest.

[7] Porcel JM, Light RW (2006). Diagnostic approach to pleural effusion in adults. Am Fam Physician.

[8] Abdelaziz AO, Hassan RN, Elghany EA (2023). Evaluation of adenosine deaminase as a diagnostic marker in tuberculous pleural effusion. Curr Respir Med Rev.

[9] Klimiuk J, Krenke R, Safianowska A (2015). Diagnostic performance of different pleural fluid biomarkers in tuberculous pleurisy. Respir Med.

[10] Bueso JF, Hernando HV, Garcia-Buela JP (1988). Diagnostic value of simultaneous determination of pleural adenosine deaminase and pleural lysozyme/serum lysozyme ratio in pleural effusions. Chest.

[11] Ferreiro L, Toubes ME, San José ME (2020). Advances in pleural effusion diagnostics. Expert Rev Respir Med.

[12] Jiang L, Li Y, Wang L (2021). Recent insights into the prognostic and therapeutic applications of lysozymes. Front Pharmacol.

[13] Ji S, Kou W, Luan P (2020). Plasma vaspin is an effective biomarker for evaluation of future cardiovascular events in patients with chest pain: a 5-year retrospective observational study. Ann Transl Med.

[14] Hussain S, Muhammad M, Qaisar O (2023). Factors affecting pleural fluid adenosine deaminase level and the implication on the diagnosis of tuberculous pleural effusion. Pak J Chest Med.

[15] Zhao T, Zhang J, Zhang X, Wang C (2024). Clinical significance of pleural fluid lactate dehydrogenase/adenosine deaminase ratio in the diagnosis of tuberculous pleural effusion. BMC Pulm Med.

[16] Charan J, Biswas T (2013). How to calculate sample size for different study designs in medical research?. Indian J Psychol Med.

[17] World Health Organization (2026). World Health Organization: Global tuberculosis report 2023. World Health Organization.

[18] Porcel JM (2018). Advances in the diagnosis of tuberculous pleuritis. Curr Opin Pulm Med.

[19] Feng C, Chan WC, Lam Y (2019). Lgr5 and Col22a1 mark progenitor cells in the lineage toward juvenile articular chondrocytes. Stem Cell Rep.

[20] Light RW (2010). Update on tuberculous pleural effusion. Respirology.

